# Merging Data Diversity of Clinical Medical Records to Improve Effectiveness

**DOI:** 10.3390/ijerph16050769

**Published:** 2019-03-03

**Authors:** Berit I. Helgheim, Rui Maia, Joao C. Ferreira, Ana Lucia Martins

**Affiliations:** 1 Logistics, Molde University College, Molde, NO-6410 Molde, Norway; Berit.I.Helgheim@hiMolde.no; 2 DEI, Instituto Superior Técnico, 1049-001 Lisboa, Portugal; rui.maia@tecnico.ulisboa.pt; 3 Instituto Universitário de Lisboa (ISCTE-IUL), ISTAR-IUL, 1649-026 Lisbon, Portugal; 4 Instituto Universitário de Lisboa (ISCTE-IUL), BRU-IUL, 1649-026 Lisbon, Portugal; almartins@iscte-iul.pt

**Keywords:** big data, data, ETL, framework, integration, knowledge, medical records, extract-transform and load

## Abstract

Medicine is a knowledge area continuously experiencing changes. Every day, discoveries and procedures are tested with the goal of providing improved service and quality of life to patients. With the evolution of computer science, multiple areas experienced an increase in productivity with the implementation of new technical solutions. Medicine is no exception. Providing healthcare services in the future will involve the storage and manipulation of large volumes of data (big data) from medical records, requiring the integration of different data sources, for a multitude of purposes, such as prediction, prevention, personalization, participation, and becoming digital. Data integration and data sharing will be essential to achieve these goals. Our work focuses on the development of a framework process for the integration of data from different sources to increase its usability potential. We integrated data from an internal hospital database, external data, and also structured data resulting from natural language processing (NPL) applied to electronic medical records. An extract-transform and load (ETL) process was used to merge different data sources into a single one, allowing more effective use of these data and, eventually, contributing to more efficient use of the available resources.

## 1. Introduction

Developments in medical research lead to an increase in the life expectancy of populations. Although this evolution leads to very positive outcomes, such as the end of some diseases (smallpox, plague, etc.) and the discovery of new approaches to several others, other challenges occur more often, such as dementia, cancer, etc. However, as medical knowledge grows and develops, other areas like computer science experience developments which can be used to support physicians in addressing these challenges. At present, healthcare providers produce and store large volumes of data, both medical and nonmedical. These data can regard drug prescriptions, treatment records, general check-up information, physician’s notes, medical information, or financial and administrative information. These data are essential not only to follow up on patients but also for management or research purposes. The knowledge that can be taken out of these data is therefore relevant for many purposes, such as service quality, by allowing the physician to access details regarding the patient that allow to improve diagnosis and decision-making. Service productivity can also benefit from this knowledge, as access to the required data is faster, letting resources increase the volume of their outcome and service control can be enhanced, by allowing disclosure of medical and performance data, among many other benefits. Data can be stored either in legacy systems or electronic medical records (EMR) [1]. EMRs are computerized medical information systems that collect, store, and display patient information [2].

It is clear that analyzing data in an integrated way can drastically improve patient quality of care and both clinical and financial outcomes, but the way in which we collect, read, integrate, understand, and leverage data remains a broken process. Data integration and consequent analysis allows full approaches in healthcare diagnosis. A diverse range of prior studies focused their effort on analyzing data to predict waiting times, based on months, weekday, weather conditions, and external events like a football match or concert, among others [3]. Natural language processing (NPL) can extract information from electronic medical registers in a semistructured way and generate additional data, like the type and number of prescriptions, date of appointment, diseases, and need for surgery, among others [4]. Data and digitalization processes allow comparative data analysis, the use of past data events allows to predict new ones, and large volumes of data can be processed with data mining and machine learning algorithms.

The format in which this information is recorded is essential because it has a direct impact on how it can be modeled to provide greater insight. Three different types of data can be found: Structured, semistructured, and unstructured [4]. Different data manipulation techniques, such as data mining (DM) and text mining (TM) or natural language processing (NLP) [5] are available, along with the associated data cleaning, data merging, and visualization approaches.

Data, information and knowledge are three commonly misunderstood concept words that are occasionally used as equals, which may be the cause of some misunderstandings [6]. The three terms are related in a pyramid/chain-like relationship [7], yet they do not represent the same concept. The complexity and understanding increase from data to information and finally to knowledge. Data are a value, like a clinical measurement, such as heart rate (for instance, 50 beats per minute) [7], or just raw data, such as a clinical narrative [6]. Information, the next level in the hierarchy, is the result of data manipulation using processes such as referential, type, purpose, relevance, and interpretation [8]; in other words, data are put into context, acquiring some meaning. Continuing the example, in the context of a small child, a heart rate of 50 bpm gives some information to a doctor about the child, yet that same information could have different meanings if an adult presents the same values [7]. The third level in the scale, knowledge, is the most informative of the three. When information is structured and organized as a result of cognitive processing [7] to offer understanding, experience and accumulated learning [8] and validation, it becomes knowledge. Thus, to recapitulate, while data are raw values, information is what is achieved when those values are put into context, and, finally, knowledge is information that is structured, organized, and processed, and may be used to improve procedures or other processes.

With the purpose of transforming data into knowledge so that medical professionals can improve the quality of the services they provide and the use of the time they have available, this work aims at developing a framework process to integrate data from different sources and make it available in a more effective way. In doing so, data from different medical records that result from a natural language processing (NPL) transformation are operated using an extract-transform and load (ETL) procedure to produce a single integrated file, allowing more effective use of that data. Addressing the goal of this research, this work starts with an analysis of the state-of-the-art in the area of data manipulation, considering the different types of data (structured, semistructured, and unstructured). Then, the methodology considered for the development of the framework is exposed. Three use cases are considered, two focused on structured data and a third one based on semistructured and unstructured data. The case studies that are presented concern the use of structured and unstructured data together and how the healthcare sector can gain value from the mixed use of these three types of data sets. The discussion is produced based on the findings from both use cases.

## 2. State of the Art

Structured data are data that are stored in a fixed schema database, and out of the three data types, they are the easiest one to manage. The most common data in this scenario are demographic (e.g., race, ethnicity, birth date), admission and discharge dates, diagnosis codes (historic and current), procedure codes, laboratory results, medications, allergies, social information (e.g., tobacco usage), and some vital signs (blood pressure, pulse, weight, height) [9]. The fact that data are stored in a structured schema makes them easier and faster to access.

There is no consensual definition of data mining (DM). For example, according to Reference [10], DM is described as “(…) the process of finding previously unknown patterns and trends in databases and using that information to build predictive models. Alternatively, it can be defined as the process of data selection and exploration and building models using vast data stores to uncover previously unknown patterns.” It can also be considered a methodology for discovering meaningful correlations, patterns and trends by sifting through massive amounts of data stored in repositories. Data mining uses a pattern recognition algorithm with a machine learning approach, as well as statistical and mathematical techniques. There is a wide range of different techniques, benefits, and areas that have been using DM, each for a specific situation. In the following sections, some case studies about DM in healthcare are reviewed to understand the diversity of techniques available, in which areas they are used, and the impact of each one of them. To facilitate the understanding on how structured data are managed and used in the healthcare sector, some cases are presented in Table 1, adapted from Reference [11,12], in which we divide the models into six areas: Classification, clustering, time series, regression algorithms, association, and hybrid model. For all of these DM types, many methods were used, showing the wide variety of techniques, applications, and possibilities. From cases studies [13,14,15,16], it is possible to access that there are many cases, in this scenario, nine out of 12, that use statistical methods as a base for classification models. On one hand, the majority of these case studies use DM techniques to train models and later apply them to predictions in many sectors, some more technical, such as cardiology [15,17,18], oncology [19], psychiatry [20], endocrinology [21], while others more focused on management, specifically quality of service [22], risk of patient rehospitalization [13], and forecasting daily bed needs [23]. On the other hand, as shown in Reference [16], predictions are not the only use of these structured data. Ravindranath explained that there are many ways to diagnose heart diseases and proposed a new decision support system for effect based on decision trees (DT) [16]. Analyzing Table 1, it can be perceived that there is a wide variety of methodologies or application of the studies that can be used. Nonetheless, it is possible to perceive that there are many studies that focus on prediction as previously stated.

### 2.1. Unstructured Data

The unstructured data of an EMR are present in clinical notes, surgical records, discharge records, radiology reports, and pathology reports [6]. Clinical notes are documents written, in free text [6], by doctors, nurses, and staff providing care to a patient, and offer increase detail beyond what may be inferred from a patient’s diagnosis codes [26]. The information contained in clinical notes may concern a patient’s medical history (diseases, interventions, among others), family history of diseases, environmental exposures, and lifestyle data [4,6]. According to Reference [27], the knowledge of said notes is retrieved “by employing domain experts to curate such narratives”, which is not practical manually. Therefore, applying an automatic way of interpretation of these clinical notes and records is of the utmost importance. As explained, DM techniques that could be applied to structure data cannot be applied to this type of data without some previous structuring (preprocessing). Instead, TM is used to prepare and process the free text data. In the next chapters, we analyze how TM tools can be used, and case studies are presented. As this type of information is represented as free text, there is no common framework, and there may be improper grammatical use, spelling errors, local dialects [5,6], short phrases, and/or abbreviations [4,6]. Due to such difficulties, data processing and analysis becomes more difficult. A great deal of this difficulty is precisely the preprocessing of the so-called free text. Natural language processing (NLP) tools and techniques are used during the preprocessing phase and have proven very useful when it comes to extracting knowledge from ERM [4,6]. NLP technology involves the ability to turn text or audio speech into encoded, structured information, based on an appropriate ontology [6]. The structured data may be used solely to classify a document or ERM in a classified system or to identify findings, procedures, medications, allergies of patients, and others [6]. Therefore, with the help of methods like NLP, it becomes possible to structure the free text and apply DM techniques. Some examples of the types of operations that are done in preprocessing are the removal of digits, anonymization, and punctuation removal, among others. Table 2 shows some studies in the area of NLP and/or TM to extract structured information. This information can be later used in DM models for prediction [27] and disease identification [28,29,30].

There are some TM techniques that are quite common, such as the removal of stop words, the lowercase treatment. Preprocessing depends on the data in hand and what that data are intended for. Table 2 shows examples of cases in which TM was used. Although the use of NLP methodologies and tools was different in all of them, the results were very satisfactory. Another significant aspect of these studies is that the unstructured data of the EMR that would not be used in some cases because it was too “wild” ended up being used and proved its usefulness.

### 2.2. Mixed Approaches

Until now, both DM and TM case studies have been shown and analyzed. While in some, only structured data were used, others only use the structured data extracted from the unstructured free text from de NLP processing used. Table 3 shows mixed approach case studies organized by year, application, reference, and methods. In these case studies, not only NLP techniques were applied, but DM techniques were also used:

- In Reference [31], topic modeling was used. It is a type of statistical modeling for discovering the abstract “topics” that occur in a collection of documents, and latent Dirichlet allocation (LDA) is an example of a topic model and is used to classify text in a document to a particular topic.
- In Reference [32], both techniques (Bayesian belief networks and decision trees (DT)) were used, and their results were compared. In other words, to detect early stages of dementia, the authors used two different techniques to assist specialists in the diagnosis of patients with clinical suspicion of dementia. It was possible to conclude that the model that used the structured data and the clustering of the texts written in free format by the physicians integrated, improved the accuracy of predictive models in all pathologies [32].
- In Reference [33], a prediction of patient admission was performed, applied to the logistic regression using five different iterations.
- In Reference [34], researchers had the objective of comparing the number of geriatric syndrome cases identified using structured claims and structured and unstructured EMR data to understand the added value of the latter. Conclusions were that results improved when combining both models. This type of fact led the authors to encourage “incorporating NLP methods to increase the sensitivity of identification of individuals with geriatric syndromes” [34].

### 2.3. Data Formats in Medicine

Recent research in the data processing [35] and biomedicine fields [36,37,38] underline the need for associating structure and information type with the datasets used in scientific projects. This information can be used to support standardization approaches and is most relevant regarding comma-separated values (CSV) or other tabular text formats where data types and structures are not enforced.

The need for preprocessing procedures for data standardization has been recently explored, in particular when considering CSV or other tabular formatted text sources. Arenas et al. [35] pointed out that skewed tabular data—with missing rows and columns—is likely to occur, specifically considering CSV and other text tabular data formats, where typically there is no metadata information. The authors proposed a framework for automatically annotating CSV-like data.

Aiming to facilitate data exchange and application interoperability through dataset reuse, this group was focused mainly on how metadata should be associated with a previously created dataset. The authors proposed the final recommendations based on the analysis of 25 case studies (http://w3c.github.io/csvw/ use-cases-and-requirements/). Regarding the scope of our work, some of the W3C recommendations should be underlined, in particular: (i) The relevance of associating syntactic and semantic information to each data column, and (ii) the relevance of defining only one data type for each column. The W3C work group suggested that the delivery of a dataset should be associated with metadata creation and sharing. Nonetheless, considering already available datasets, the workgroup stressed that the publication of metadata could benefit the user community and can be performed by an independent party, in addition to the dataset provider. The group argued that this possibility might enable the user community to benefit from each other’s efforts when it comes to dataset understanding and exchange.

### 2.4. Data Processing in Medicine

Ongoing research based on data collected during health care service delivery is increasing patient-centered knowledge [37]. However, the quality of EMRs is considered as not complying with research standards [36], which pushes research teams to overcome data quality problems using data preprocessing implementations. These implementations are frequently seen as near ad hoc solutions [35,37], not following any standardized data preprocessing approach. Wu et al. [39] published an extensive analysis of the impact of data preprocessing, modeling, and mining in the biomedicine context. These authors analyzed and described problems in structured and unstructured data, including data in EMRs. One of the work’s main conclusions was the need for data integration and interpretation guidelines as a factor that enables better prediction and therapeutics. Feder [37] focused mainly on structured information in EMRs, underlining the frequently missing information regarding data quality dimensions (like consistency or completeness, for example). The author recommended three general principles to be followed in EMRs research works: (i) The use of metadata definitions (referred to as data dictionary) for every dataset used, (ii) the use of statistical methods to deal with data quality issues (e.g., missing data) and finally, (iii) the generation of a report containing relevant data quality information (e.g., the proportion of missing data found, number of variables removal, and the number of transformations applied). Back in 2013, concerned with the lack of standardized data preprocessing approaches in data-driven biomedicine research, Weiskopf and Weng [40] analyzed 95 data-driven biomedicine research articles searching for the data quality dimensions most frequently referred by authors. This work was used by Reimer et al. [38] in their proposal of a six-step framework for data quality assessment in longitudinal registries (i.e., datasets built on heterogeneous data sources). Although the authors were not focused on analyzing data preprocessing methods, they concluded that associating metadata with datasets [38] is a useful factor when considering dataset reuse or replication.

## 3. Work Methodology Proposed

**Figure 1:**
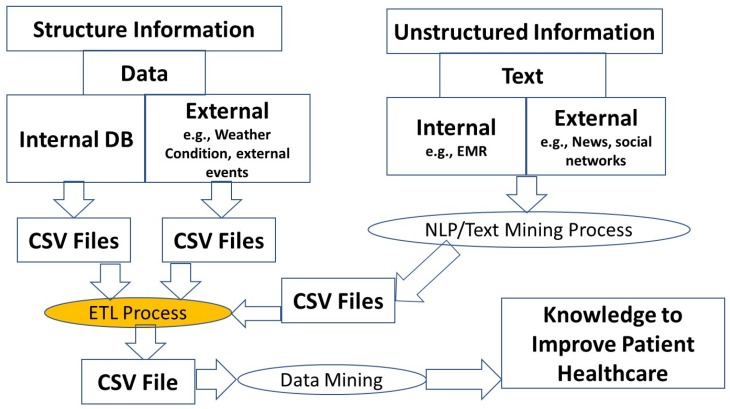
Methodological paths of the research.

The main goal of this research was the definition of an ETL process for the creation of a single data set to improve the mining, prediction, and visualization process of data from several sources. The main idea is illustrated in Figure 1.

Recommendations from Arenas et al. [35] were used in our research, as well as from the World Wide Web Consortium (W3C) [41].

**Figure 2:**
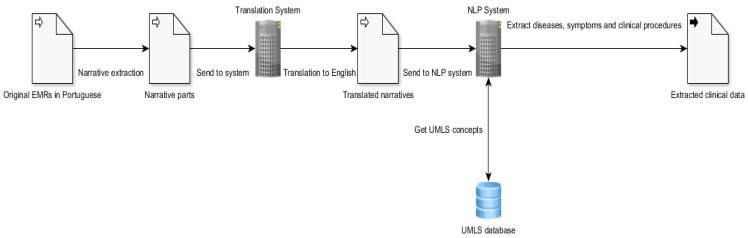
A high-level view of the whole pipeline system.

In order to extract clinical information from any EMRs, a whole system based on open-source modules is coupled together. Firstly, a translator is required to translate the EMRs’ clinical narratives from any language to the English language, with reasonable performance. Secondly, we need an open-source NLP system responsible for applying information extraction techniques and performing the clinical information extraction from the EMRs’ narratives (see [6]). An ontology unified medical language system (UMLS) to help biomedical vocabularies and standards is required to enable interoperability between computer systems. This UMLS is a repository of biomedical vocabularies developed by the US National Library of Medicine, containing more than 2 million concepts and 8 million concept names, some of them in different languages than English. A high-level depiction of the pipeline system we aim to build in this work is shown in Figure 2, and for more details and achieved results, see Reference [6].

The output of this process is data extracted of diseases, symptoms, and clinical procedures, from the EMRs’ narratives, outputting a file with all of the extractions concerning those domains. These data are transformed into a CSV file to be able to be merged with structured data. Research teams commonly need to validate, clean or transform data contained in CSV input datasets so that they can be used in experimental setups. For that reason, researchers frequently implement ad-hoc preprocessing routines not completely specified in published papers, which can make work reproduction and validation difficult. The purpose of this work is to merge different source files, using as a validation approach the data in CSV formats. The use of heterogeneous data sources in data-driven scientific projects frequently requires a preprocessing phase where input data are interpreted, validated, transformed, and saved into an output data format that is typically different from the input data format. The results of each project seriously depend on the quality of data used in the experiments, regardless of their purpose (statistical analysis, knowledge discovery or other). The data preprocessing phase is crucial. Despite not having any data type information, CSV files are one of the simplest and most extensively used formats when it comes to data exchange [36].

### Data Integration Using ETL Process

**Figure 3:**
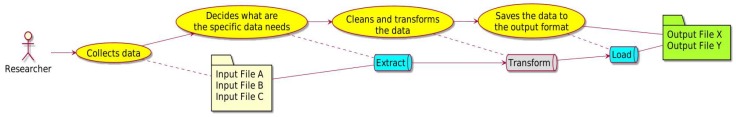
Research data preprocessing viewed as an extract-transform and load (ETL) process.

In order to address the lack of data preprocessing standardization in scientific projects, we propose to model the data preprocessing phase as an extract-transform and load (ETL) process. ETL derives from data warehousing and covers the process of how data are loaded from the source system to the data warehouse. A typical data preprocessing phase is thus composed of the following three phases: (i) Extract available data (extract), (ii) transform and clean data (transform), and (iii) store the output data in an output repository (load). These phases are preceded by the data source selection, typically a set of files or a database. Figure 3 illustrates the process. More detailed insight of each of the three phases is provided:

1) The extract process is responsible for the data extraction from the source system or database and makes it accessible for further processing or management process. In health care, this process needs to deal with data privacy, and most of the extract process has an anonymization process associated. At this point, the researcher decides which data make sense to use.
2) The transform is a process based on a set of rules to transform the data from the source to the target. In the current research, a new approach using semantics is proposed for this phase. This can be a complex process taking into account different dimensionalities cases, and it needs to assure that all variables are in the same units so that they can later be joined and a clean process can be conducted. The transformation step also requires joining data from several sources, generating aggregations, surrogate keys, sorting, deriving newly calculated values, and applying advanced validation rules.
3) The load process merges all data into a target database (output data).

**Figure 4:**
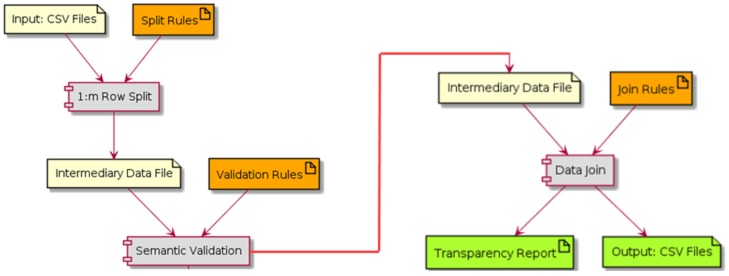
Detail of the Transform phase of the proposed (E3TL) approach that is applied to comma-separated values (CSV) input files.

The processed (output) data can then be used by data analysis and knowledge discovery algorithms. We structure our work in the transformation phase by dividing it into three sequential steps (E3TL). For each of these steps, there is an associated auxiliary configuration file. The first two steps are specific for each input file having its own specification and intermediary output file. The third and final step is unique and generates the final CSV output files. In more detail, the three E3TL steps are as follows (see Figure 4):

(1) Split one row into many (1 : m row split). Input rows are split in a 1 : m transformation, in order to have just one row per patient examination (instead of one row containing multiple patient examinations). At this point, only the relevant input fields are kept. The specification of row splitting and field selection is made through an auxiliary file called split rules. In this file, each selected field of the input CSV file is associated to a type and eventually to a structure (e.g., a specific date format to be used such as ‘YYYY-MM-DD’, for ‘2019-01-23’).
(2) Semantic validation. At this step, the resulting data files of 1:m row split are submitted to semantic validation, where the rules defined in the auxiliary file semantic rules are applied. These rules are based on domain knowledge and aim at validating specific domain constraints, like thresholds or non-empty fields in specific columns;
(3) Data join. At this point, data is joint by applying the rules defined in the last auxiliary file, named join rules. This file describes which fields should be present in the output files and which will be used as keys when joining the rows from each file. In addition to the output data files, a file named transparency report is generated. It includes statistical information about operations applied during the transformation process.

In order to validate our methodology, we applied it to a biomedical dataset, specifically to an amyotrophic lateral sclerosis (ALS) dataset produced by neurologists from Hospital de Santa Maria, in Lisbon, Portugal. This dataset contains information about ALS patients, including personal and medical examination information that was previously anonymized. In our experimental implementation, all transformations were coded in Python and the supporting files specified using Javascript notation (JSON) notation. Our experimental implementation of the E3TL methodology was also coded in Python. JSON was also used for auxiliary files specification. The implementation is available either as a Jupyter Python notebook and the equivalent Python source [https://github.com/ruifpmaia/e3tl]. The python file is imported as a module, and then each E3TL transformation can be invoked as a function.

## 4. Description of the Case Study

Under the scope of our work, we analyzed Amaral et al.’s [42] and Carreiro et al.’s [43] work on amyotrophic lateral sclerosis (ALS) disease evolution prediction, specifically on respiratory failure prediction in ALS patients. Our work focuses on proposing a new E3TL methodology based on the researcher’s data preprocessing approach, namely on row split (the authors refer it as execution split) and other frequently used data validations and transformations. For an extensive understanding of the preprocessing routines used by the authors, we also analyzed their code implementation in Java programming language and the obtained output files. The same ALS dataset was used in both experiments, made available for research purposes by Hospital de Santa Maria, a main Portuguese Hospital located in Lisbon. The model was applied to an ALS dataset produced initially as a Microsoft Excel spreadsheet format (XLS) by neurologists of Hospital de Santa Maria, containing information on 495 patients. The XLS file included 25 worksheets which were exported to 25 CSV files. Our approach generated, as expected, five output files matching the predefined output formats and a transparency report, statistically describing the E3TL process with information such as the number of input and output fields, the applied validations, and the number of input and out rows in each step. Data fields are delimited by one constant character and values can be simple (as integers, for example) or complex, like dates, floats or text comments. Each patient is uniquely identified by a number that is used across the different files as an identifier. Table 4 describes the data stored in the dataset in generic terms. In order to facilitate dataset processing, each worksheet was exported to a different CSV file. The dataset contains two classes of data: (a) Static, also referred to as administrative data, and (b) temporal data. Static data are independent of medical examinations and applied treatments and include personal information like age, gender or family history. Dynamic data are collected over multiple examinations and capture the results of the applied treatments. Each medical examination is composed of multiple clinical tests. These tests, associated with the same examination, may span several days or weeks. According to References [42,43], only 6 of the 25 input worksheets were used in the experiments, namely: Demographics, ALS-FRS, ALS-FRS1, NPO, Phrenic, and RFT. Demographics contains static patient information and family history, while ALS-FRS, ALS-FRS1, NPO, and Phrenic contain dynamic information obtained during medical examinations. 

Table 5 and Table 6 are examples of tabular data formats stored in the input worksheets. Table 6 contains the amyotrophic lateral sclerosis functional rating score (ALS-FRS) examination results. It is an example of dynamic data in which each line is associated with a patient, containing the results of multiple ALS-FRS examinations. The example shows how fields (Date, FeatureA, and FeatureB) are repeated in the same line, representing the values of two tests performed in different dates on the same patient.

First, in 1:m row split, input rows are split. For example, each row of the ALS-FRS.csv file is analyzed, and if there is more than one medical examination per row, then it will be transformed into multiple rows according to the parameters in the auxiliary file named split rules. Each row field is parsed and formatted according to parameters also defined in the auxiliary file. For example, dates can be parsed and reformatted, or decimal conventions (decimal marks: ‘.’ or ‘,’, for example) can be normalized. This step will generate intermediary output files. The semantic validation step is applied to the intermediary output files produced by the first step. In this step, a significant number of rows might be removed due to validation of missing data, erroneous or unexpected values. These validations are parameterized by the semantic rules file. Mandatory fields found as missing or values under a required threshold, for example, might result in row deletion. Again, this step will produce new intermediary output files. The last step, data join towards the load process, consists of executing join procedures that will generate the final output files. All available fields from the intermediary output files (generated by the last step, semantic validation) can be used to form the final output file according to the parameters specified in the join rules auxiliary file. Addressing a double validation of E3TL, additional to a full verification against the descriptions in References [42,43], we checked the output files generated by our methodology with the ones received by authors implementations. The next three subsections detail each of the E3TL steps and the available parameters in each auxiliary configuration file.

### 4.1. E3TL First Step—The 1: m Row Split

**Figure 5:**
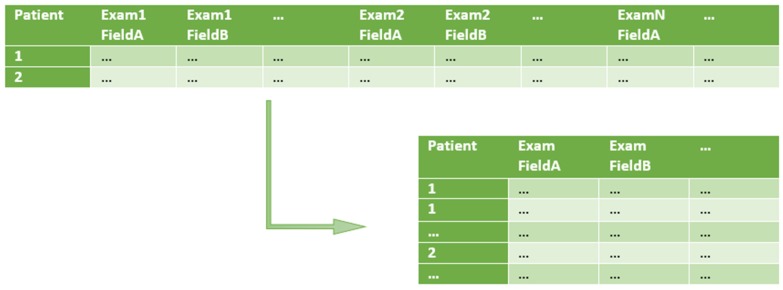
1:m row split example.

The first step of our methodology focuses on input files containing medical examinations. Each input file should have its own field mapping auxiliary file. This step splits each row of an input file into multiple rows whenever there is more than one medical examination per row. The resulting rows will only have the fields specified in the auxiliary configuration file metadata map (see Figure 5). Each field to be kept is associated with a data type and a specific structure (e.g., a date format ‘YYYY-MM-DD’). The 1:m row transformation depends on the knowledge about the structure of input files (Figure 5 exemplifies a row normalization transformation). Typically, it will not be applied for static data, such as a demographic information file. In the Santa Maria ALS dataset, each medical examination file contains one row for each patient, each one including multiple medical examinations. The same set of fields is repeated (as columns) through all exam iterations in the considered file. This step’s auxiliary file, identified as aplit rules, describes the field structure of the input and intermediary output file, specifying the fields that should be kept. Each field to be kept is associated with a metadata description, defining its type and format (when needed). This mapping operation is applicable whether there are multiple exams per row or just one exam per row. The definition of the split rules parameters requires knowledge about the field structure of the input data file. It specifies the fields to be present in the rows of the intermediary output file. This auxiliary file contains the following information:

- **OutFile**—The intermediary output file generated by the field mapping step;
- **ExamFeatures**—The number of fields per examination. This variable supports the indexing of each input field with the correct offset;
- **ExamCountPerRow**—The number of examinations per row. In the ALS-FRS.csv file, each row is associated with a specific patient. Therefore, it includes multiple examinations per row;
- **OutputFeatures**—The number of fields of the output table;
- **FeatureMapping**—The list of mappings between the column index of the selected input fields and the desired column output index;
- **InputIdx**—The index of a specified column in the input file;
- **output is**—The position of the field in the output intermediary file;
- **StaticIdx**—There are static and non-static based indexes. A static input column index is not dependent on the number of examinations per row. Differently, the “Date” field, with a non-static index, will be retrieved using the offset: InputColumnIndex = (exam number ∗ExamFeatures) + InputIdx where ExamNumber <= ExamCount;
- **Name**—The name to be used in the output file;
- **Type**—The data type to be used. Depending on the data type, different parsing routines will be used, either for multiple date formats, or different decimal notations, for example;
- **Format**—The output format of non-basic types to be used. For dates, for example, ‘YYYY-MM-DD’, for ‘2019-01-23’.

Regarding type and formatting, the 1:m row split can parse different multiple date input formats and decimal symbols in order to convert them to the selected output format. At the end of field mapping step, an intermediary file is generated having just one patient examination per row.

### 4.2. E3TL Second Step—The Semantic Validation

The second step of the transformation phase applies data domain validations to an intermediary file generated by a 1 : m row split. Like in the previous step, there is different specification of the semantic validation step for each file, as well as a different validation rules auxiliary file. This step runs three types of validations: (1) Missing values to verify if a field is empty; (2) threshold validation, to check if a value is under or above a specified threshold; and (3) regular expression validation, to analyze a value against a regular expression (for example, to validate if a field contains only integer values: ^\d+$). These three types of validations were shown to cover the needs of the case study. Taking the ALS-FRS.csv file as an example, the validation and filtering of exam rows depended on the verification of patient ID, as the first output field, with the “InputIdx” : 0. It should be simultaneously non-null (“NotNull” : true) and a positive integer, represented through the regular expression "RegEx": "^[0–9]+$". An example of the validation rules of an intermediary file (ALSFRS.csv) obtained from the processing (by field mapping) of the ALS-FRS.csv input file. The available parameters for the validation rules auxiliary file are the following:

- **OutFile**—The intermediary output file generated by the semantic validation step;
- **Semantic Rules**—The list domain validations that should be applied to a specific field;
- **InputIdx**—The index of a specified column in the input file. NotNull requires a non-null value;
- **RegEx**—Verify if the value of the field maps to the specified regular expression;
- **Threshold**—Verify if the value of the field is over the specified threshold (only applicable to integer or double fields).

### 4.3. E3TL Third Step—Data Join

The last step of the transformation phase is a join transformation where fields from the intermediary output files generated by the semantic validation step are possibly merged. According to the rules defined in the associated auxiliary file join rules, this step executes an Inner Join operation using the specified key. The result will be the generation of an output file with the specified fields. The available parameters of the join rules auxiliary file are the following:

- **OutFile**—The final output filename;
- **FeatureMapping**—The list of fields that should be present in each row of the output file and their relation to the input fields (from the intermediary files). Typically, each row includes dynamic (exams) and static information (like patient date of the birth, for example);
- **InputIdx**—The index of the input field on the auxiliary input file.
- **InputFile**—The auxiliary input filename;
- **Name**—The final output field name;
- **Type**—The output field data type;
- **Format**—The output field format for non-basic types (like dates, for example);
- **JoinKey**—Indicates if the field is part of the Join key.

Table 7 presents an example of the ALSFRS file joined with the NIV (non-invasive venthilation) field retrieved from the Demographic file.

## 5. Outcomes from the Case Study

Our methodology produced output files with the expected data and structure according to the specified E3TL process. The output files generated for the Santa Maria dataset include dynamic and static information. The dynamic information was retrieved from the original files containing data of medical examinations (i.e., NPO, ALSFRS, Phrenic, and RFT) and was later joined with static information from the demographics file. The output result was stored in five different files and represented in Table 8, Table 9, Table 10 and Table 11 (the demographics output table was split in three, due to its size). Results from the three E3TL steps were as follows:

(a) 1 : m row split execution example. The function receives two variables as arguments: One with the name of the CSV input data file and the second with the name of the JSON specification auxiliary file. As output, the function returns the name of the intermediary CSV output file.

(b) Semantic validation execution example. The function receives two variables as arguments: one with an intermediary CSV data filename generated by the previous step, and the second with the name of the JSON specification auxiliary file. As output, the function returns the name of the intermediary CSV output file.

(c) Data join execution example. The function receives as arguments the JSON specification associated auxiliary file. As output, the function returns the name of the final CSV output file.

The process generated a transparency report with the statistical description of input and output datasets and the number of operations executed in each phase of E3TL. The number of transformations applied and missing values found, for example, are registered in this file. Table 12 (a), (b), (c) presents the collected information.

## 6. Other Application Cases

**Figure 6:**
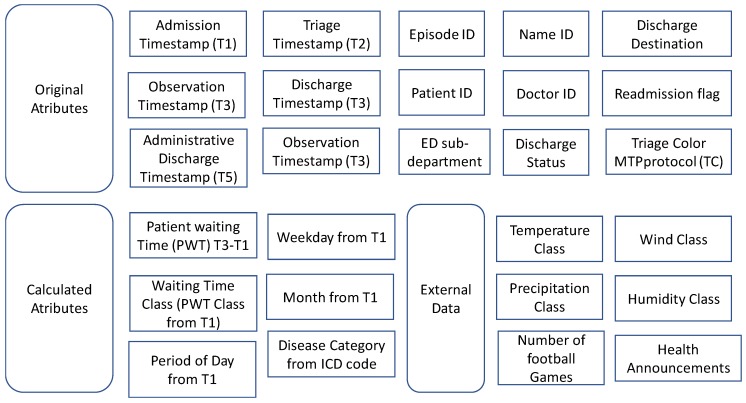
Data integrated from a hospital in Lisbon using the proposed ETL process.

Some procedures with small changes on Python scripts were applied to urgency data from a hospital in Lisbon. These data include registers from 1 January 2013 to 31 December 2017. The original data were enriched with external data, like weather conditions and events. The ETL process was applied to generate a greater data file (see Figure 6) that allows improvements in the mining and prediction processes. This integration allowed the prediction of waiting times based on the day of the week, on the month, weather conditions and correlations with special seasons, and events, among others. For details, see Reference [43,44].

**Figure 7:**
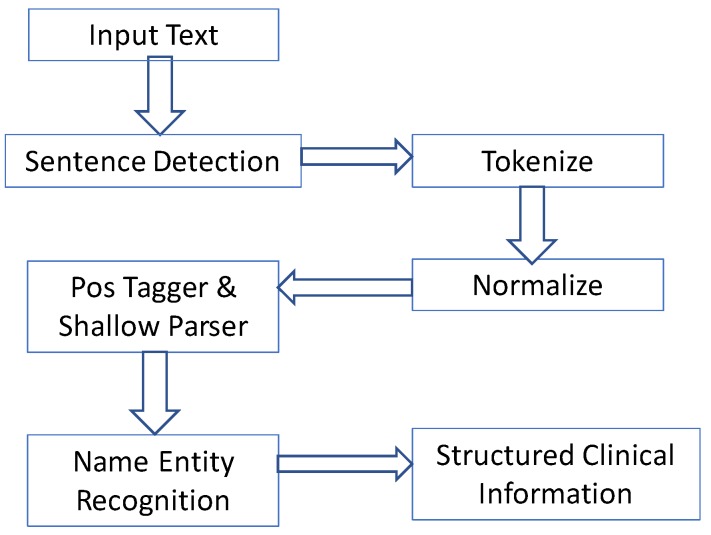
Schema of developed components involved in the text processing.

In another use case, these data were integrated with the output of NLP over EMR [6]. A schema of all the components involved in the text processing is shown in Figure 7. This schema was applied to 5255 EMRs from a Portuguese hospital. Once all the extracted clinical information is structured and gathered in a CSV file, it is possible to apply the ETL process and create a single CSV file that can be loaded for the data mining process in open sources or commercial platforms. Results of this are available in Reference [6].

The evaluation of the NPL system was performed based on standard metrics calculated for 75 of the 5255 EMRs. These standard metrics are precision, recall, and F1 score. Two healthcare practitioners from the hospital manually annotated the clinical terms present in 75 EMRs in order to establish a gold standard for this evaluation. The evaluation showed that the NPL system coupled together in this research has a precision of 0.75, recall of 0.61, and an *F*1 score of 0.67.

## 7. Discussion

The main drawback of the CSV format is the fact that it does not enclose data and information type for the stored fields. This problem exponentially escalates when dealing with large datasets, updated by multiple users or organizations and using different standards for information representation. Typically, research teams working on data-driven scientific projects focus on data analysis and knowledge discovery algorithms, trying to obtain, for example, the best prediction models and results. They implement ad hoc data preprocessing routines based on their domain knowledge for data validation and transformation operations. Preprocessing routines are commonly not detailed in published works that are mostly focused on reporting the implemented data analysis techniques and obtained results. Little attention is given to preprocessing standardization. Considering tabular formats like CSV, this lack of documentation or incomplete description can be a relevant issue hurdling work assessment and reproduction. Without ensuring reproducibility, it may not be possible to re-execute part of or an entire scientific work, thus restraining its validation or further development.

We propose improvements in the area of tabular data integration and data preprocessing in the medical area. A new ETL methodology—E3TL—was proposed based on CSV file preprocessing in data-driven scientific projects aiming at standardized procedures for data integration, cleaning, and transformation.

Our approach has the advantage of defining a comprehensible and systematic ETL process for CSV preprocessing. It also integrates three primary vectors concerning data processing and exchange (or reuse) transparency: (i) Metadata declarations as a basic structure for data preprocessing; (ii) systematic data validation definitions; (iii) integrated transformation, validation, and filtering metrics summarized in a transparency report. By including metadata information in E3TL, we aimed at facilitating interpretation and sharing of the original dataset knowledge, following W3C recommendations.

The results from our research can improve the quality of the service provided to the patients at the hospital as data are integrated and knowledge is available to medical professionals to gain a broader and more complete picture of the patient they are treating. In parallel, by having the data available and transformed into knowledge, they can lead to faster and more accurate diagnosis, which allows’ medical personnel to become more productive. From a more managerial perspective, this type of integration and the knowledge it makes available can also be used, for instance, for the prediction of waiting times [43], allowing crossing it with external information, like weather conditions or big events.

Applying the proposed approach to other use cases and datasets may lead to refinements and further developments of the E3TL model, including the incorporation of more detailed statistic processes. This approach can be generalized to different cases, as long as the baseline conditions are verified. Generalization to other cases will require adjustments to the specificities of those cases. Nonetheless, the overall framework is applicable.

## 8. Conclusions

New achievements regarding the availability of knowledge from data can be performed with the merge of different data sources using as input structured and unstructured data. This is not only very positive for the hospitals and healthcare centers but, and most importantly, for the patients. Considering that the main objective of this field is offering healthcare to those in need or, in some scenarios, the best quality of life possible, by using structured and unstructured data separately, results can be obtained as shown. This mixed approach can be used not only in technical areas but also in operational ones. For example, through better disease prediction models, earlier diagnostics can be achieved, allowing a more adjusted medication to the patients and eventual avoidance of more acute health states. It can also be used to forecast seasonality and understand what sector of the hospital would be more requested in each season to improve the allocation of staff. 

Data integration and manipulation allow DM processes to be conducted using enormous data volumes to anticipate problems and/or solve them at their early stages, improve diagnosis and treatment because more data and cross-check functions are available and, in general, improve the quality of health services and patients’ quality of life.

One paramount issue in DM is that data can be spread across many entities (hospital, government, and others). The integration that results from this approach allows to create new data sets with more information, and new knowledge can be generated to improve healthcare. Further, this integration of different data from the hospital database with ERM and external data also allows to improve management procedures based on the outputs from the data mining process. 

In this approach, the privacy issue was overcome with data anonymization. Data were used with the permission of the hospital.

## Figures and Tables

**Table 1:** Example of data mining (DM) case studies from 2013–2016.

Model	Methods	Application	Sector	Ref	Year
Hybrid Model	NN and GA	Heart disease risk prediction	Cardiology	[17]	2013
Hybrid Model	K-means and clustering technologies	EMR data processing	Management	[24]	2014
Time Series Mining	Time series mining	Risk prediction of heart disease	Cardiology	[18]	2014
Association Rule Mining	Association rule mining	Diabetes risk prediction	Endocrinology	[21]	2015
Classification	Dynamic classification and hierarchical model	Re-hospitalization risk prediction	Management	[13]	2015
Classification	Multi-classifier method	Noncommunicable disease prediction	Epidemiology	[14]	2015
Classification	Support Vector Machine	Intensive Care units (ICU) risk prediction	Cardiology	[15]	2015
Classification	Decision Trees	Decision Support Systems	Management	[16]	2015
Clustering	Dynamic feature selection	Quality of medical service	Quality of Service	[23]	2015
Regression Algorithm	Ordinal regression framework	Suicide risk prediction	Psychiatry	[20,21]	2015
Time Series Mining	Time series mining	Risk prediction of colorectal cancer	Oncology	[19]	2015
Regression Algorithm	Multivariate logistic regression	Analysis of Cardiac Surgical Bed Demand	Management	[25]	2016

**Table 2:** Examples of text mining (TM) case studies from 2015–2018.

NLP Methods	Methods	Application	Reference	Year
Stemming, stop words, numbers and dates removed	Logistic Regression	Detection of Adverse Childhood Experiences	[28]	2015
Stop words, lowercase, (IDF) Inverse Document Frequency	Support Vector Machine (SVM)	Identification of fall-related injuries	[29]	2015
Self-Developed Algorithm		Coronary artery disease risk assessment	[30]	2015
Tokenization, Term Frequency-Inverse Document Frequency (TF-IDF), Removal of Stopwords	Forward Neural Network with Back Propagation	Automatic diagnosis prediction	[27]	2016
		Extracting	[6]	2018

**Table 3:** Example of mixed approach case studies 2015–2018.

Methods	Application	Sector	Ref	Year
Topic Modeling (LDA)	Identify Related Patient Safety	Events Security	[31]	2015
Bayesian Belief Networks and DT	Diagnosing Early Stages of Dementia Psychiatry	Psychiatry	[32]	2018
Logistic Regression	Predict Hospital Readmissions	Cardiology	[33]	2018
Clustering	Geriatric Syndrome Detection	Geriatric	[34]	2018

**Table 4:** Dataset description.

Characteristics	Description/Value	Comments
Data Source	Hospital Santa Maria, Lisbon, Portugal	
Data Format	Microsoft Excel Spreadsheet (xls)	Binary Format
Number of Sheets	25	Atrophy, Blood, Cramps, DeltoidEMG, Demographics, Diagnostic EMG, EPO-VGF, Fascic, Medication, MRC, NIV, NPO, PEG, Phrenic EMG, Reals, Reflexes, RFT, SCMEMG, SNIP, Spasticity, Treadmill
Static Information	Demographics (1 worksheet)	71 field of static information about each patient
Temporal Information	24 worksheets	
Number of Patients	495	

**Table 5:** ALSFRS input table using a comma-separated values file.

Case	Date	Feature A	Feature B	Date	Feature A	Feature B	Date
1	28 June 2010	not done	2.5	10 June 2011	10		
2	5 November-2010	50	N	28 February 2011	2	3.5	12 June 2011
3	10 November 2006	12,3	12.5	11 December 2006	3	2,3	

**Table 6:** Demographics input table using a comma-separated values file.

Name	Birthday	Gender (1-Male; 2-Female)	Height (m)	Weight before 1st Symptoms (kg)
1	6 June 1951	1	1.69	73
2	30 June 1951	1	1.80	58
3	3 January 2010	1		
4	1 August 1927	1	1.65	
5	27 August 1941	1	1.57	
6	5 December 1940	2	1.58	57

**Table 7:** ALSFRS output table, generated using ASLFRS (dynamic) and demographics (static) file information.

Name	Date	ALS-FRS	ALS-FRS-R	ALS-FRSb	R	NIV
1	28 June 2010	37	44	12	10	0
1	6 October 2010	37	44	12	10	0
1	5 January011	37	44	12	10	1
1	5 May 2011	37	45	12	12	1
1	6 July 2011	38	46	12	12	1
10	19 May 2003	29	37	12	12	0
10	4 August 2003	30	38	12	12	0
10	3 November 2003	24	32	12	11	0

**Table 8:** RFT output table; generated using RFT and demographics (NIV—non-invase venthilation) original input information, Forced Vital Capacity (FVC), Maximal Inspiratory and Expiratory pressures (MIP/MEP), partial gas concentrations (P0.1, PO2 and PCO2), values of mean (or under 90%) oxygen saturation (SpO2mean and SpO2 < 90%)

Name	Date	%VC	%FVC	%MIP	%MEP	%P0.1	PO2	PCO2	Weight	NIV
1	17 May 2010	85	86	29	52	64	84	43	73	0
10	12 May 2003	95	98	89	1,13	1,13	89	39	79	1
101	09 December 2004	1,06	1,1	69	1,21	61	91	35		1
102	25 August 2004	38	40	23	25	30	70	63	73	0
104	04 August 2005	32	32	19	36	3,1	88	36	46	0

**Table 9:** NPO output table; generated using NPO and demographics (NIV) original input information.

Name	Date	SpO2mean	SpO2min	SpO2 < 9 0%	Disp 4%	Disp h < 4%	Disp3	Disp h < 3%	Pattern	NIV
**1**	7 July 2010	95.56	92	0	5	0.96	10	1.92	1	1
**1**	4 April 2011	96.33	90	0	29	3.61	44	5.48	3	1
**1**	7 January 2003	94.25	89	0.06					3	0
**10**	12 February 2003	94	85	0.71	23	2.57	51	5.69	3	0
**10**	30 January 2004	94.94	85	0.35	29	2.42	56	4.67	3	1

**Table 10:** Phrenic output table; generated using phrenic and demographics (NIV) original input information.

Name	Date	PhrenMeanLat	PhrenMeanAmpl	PhrenMeanArea	NIV
1	7 July2010	8	1	3	1
10	2 December 2003	9	0	2	0
100	1 January 2004	8	1	3	0
100	16 July 2004	7	1	3	1

**Table:** **(a)** **Part one**

Name	Date	ALS-FRS	ALS-FRS-R	ALS-Frsb	R	Name_1	Gender	BMI	MND	Age At Onset
1	28 June 2010 00:00:00.000	37	44	12	10	1	1	25.6	2	59
1	6 July 2011 00:00:00.000	38	46	12	12	1	1	25.6	2	59
10	10 May 2003 00:00:00.000	29	37	12	12	10	1	26.6	2	73
10	4 August 2003 00:00:00.000	30	38	12	12	10	1	26.6	2	73
10	3 November 200300:00:00.000	24	32	12	11	10	1	26.6	2	73

**Table:** **(b)** **Part 2**

Name	El Escorial Reviewed Criteria	Onset Form	Envolved Segment—1st Symptoms	Evolution Pattern	Name
1	pro	1	LL	RLL- LLL	1
1	pro	1	LL	RLL- LLL	1
10	pos	1	UL	LUL-LLL-RUL-RLL-R	10
10	pos	1	UL	LUL-LLL-RUL-RLL-R	10
10	pos	1	UL	LUL-LLL-RUL-RLL-R	10

**Table:** **(c)** **Part 3**

Name	1st Symptoms - 1st Visit	Alive\_Death\_Disappeared	Date of Death\_Disappearance	NIV
1	18.1	0	0	
1	18.1	0	0	
10	28.3	1	20 December 2004	0
10	28.3	1	20 December 2004	0
10	28.3	1	20 December 2004	1

**Table:** **(a)** **1 : m Row Split**

Input File Names	Input Entries	Output Entries	Input Fields	Output Fields	Type Erros	Format Errors	Value Errors	Total Errors
demographics.csv	616	481	71	13	2	15	5	22
als-frs.csv	502	780	243	6	122	25	4113	4260

**Table:** **(b)** **Sematic Validation**

Input File	Input Entries	Output Entries	Regex Exclusions	Threshold Exclusions	Missing Value Exclusions	Total Exclusions
demographics.csv	481	458	6	12	5	23
als-frs.csv	780	725	6	25	24	55

**Table:** **(c)** **Data Join**

Output File	Input Entries	Output Entries	Missing Key Exclusions
demographics.csv	458	458	0
als-frs\_with\_niv.csv	725	725	24
